# Clinical and Epidemiologic Features of *Cryptosporidium-*Associated Diarrheal Disease Among Young Children Living in Sub-Saharan Africa: The Vaccine Impact on Diarrhea in Africa (VIDA) Study

**DOI:** 10.1093/cid/ciad044

**Published:** 2023-04-19

**Authors:** M Jahangir Hossain, Helen Powell, Samba O Sow, Richard Omore, Anna Roose, Joquina Chiquita M Jones, Syed M A Zaman, Henry Badji, Golam Sarwar, Irene N Kasumba, Uma Onwuchekwa, Sanogo Doh, Alex O Awuor, John B Ochieng, Jennifer R Verani, Jie Liu, Sharon M Tennant, Dilruba Nasrin, Leslie P Jamka, Yuanyuan Liang, Stephen R C Howie, Martin Antonio, Eric R Houpt, Karen L Kotloff

**Affiliations:** Medical Research Council Unit, The Gambia at the London School of Hygiene & Tropical Medicine, Banjul, The Gambia; Department of Pediatrics, Center for Vaccine Development and Global Health, University of Maryland School of Medicine, Baltimore, Maryland, USA; Center for Vaccine Development and Global Health, University of Maryland School of Medicine, Baltimore, Maryland, USA; Centre pour le Développement des Vaccins du Mali (CVD-Mali), Bamako, Mali; Center for Global Health Research, Kenya Medical Research Institute, Kisumu, Kenya; Department of Pediatrics, Center for Vaccine Development and Global Health, University of Maryland School of Medicine, Baltimore, Maryland, USA; Center for Vaccine Development and Global Health, University of Maryland School of Medicine, Baltimore, Maryland, USA; Medical Research Council Unit, The Gambia at the London School of Hygiene & Tropical Medicine, Banjul, The Gambia; Medical Research Council Unit, The Gambia at the London School of Hygiene & Tropical Medicine, Banjul, The Gambia; Medical Research Council Unit, The Gambia at the London School of Hygiene & Tropical Medicine, Banjul, The Gambia; Medical Research Council Unit, The Gambia at the London School of Hygiene & Tropical Medicine, Banjul, The Gambia; Center for Vaccine Development and Global Health, University of Maryland School of Medicine, Baltimore, Maryland, USA; Department of Medicine, University of Maryland School of Medicine, Baltimore, Maryland, USA; Centre pour le Développement des Vaccins du Mali (CVD-Mali), Bamako, Mali; Centre pour le Développement des Vaccins du Mali (CVD-Mali), Bamako, Mali; Center for Global Health Research, Kenya Medical Research Institute, Kisumu, Kenya; Center for Global Health Research, Kenya Medical Research Institute, Kisumu, Kenya; Division of Global Health Protection, US Centers for Disease Control and Prevention, Nairobi, Kenya; Division of Infectious Diseases and International Health, Department of Medicine, University of Virginia, Charlottesville, Virginia, USA; School of Public Health, Qingdao University, Qingdao, China; Center for Vaccine Development and Global Health, University of Maryland School of Medicine, Baltimore, Maryland, USA; Department of Medicine, University of Maryland School of Medicine, Baltimore, Maryland, USA; Center for Vaccine Development and Global Health, University of Maryland School of Medicine, Baltimore, Maryland, USA; Department of Medicine, University of Maryland School of Medicine, Baltimore, Maryland, USA; Center for Vaccine Development and Global Health, University of Maryland School of Medicine, Baltimore, Maryland, USA; Department of Medicine, University of Maryland School of Medicine, Baltimore, Maryland, USA; Center for Vaccine Development and Global Health, University of Maryland School of Medicine, Baltimore, Maryland, USA; Department of Epidemiology and Public Health, University of Maryland School of Medicine, Baltimore, Maryland, USA; Medical Research Council Unit, The Gambia at the London School of Hygiene & Tropical Medicine, Banjul, The Gambia; Medical Research Council Unit, The Gambia at the London School of Hygiene & Tropical Medicine, Banjul, The Gambia; Division of Infectious Diseases and International Health, Department of Medicine, University of Virginia, Charlottesville, Virginia, USA; Department of Pediatrics, Center for Vaccine Development and Global Health, University of Maryland School of Medicine, Baltimore, Maryland, USA; Center for Vaccine Development and Global Health, University of Maryland School of Medicine, Baltimore, Maryland, USA; Department of Medicine, University of Maryland School of Medicine, Baltimore, Maryland, USA

**Keywords:** diarrhea, *Cryptosporidium*, prevalence, severity, children

## Abstract

**Background:**

As part of the Vaccine Impact on Diarrhea in Africa (VIDA) Study, we examined the prevalence, clinical presentation, and seasonality of *Cryptosporidium* in children to understand its relative burden after the introduction of rotavirus vaccine.

**Methods:**

VIDA was a 3-year, age-stratified, matched case-control study of medically attended acute moderate-to-severe diarrhea (MSD) in children aged 0–59 months residing in censused populations at sites in Kenya, Mali, and The Gambia. Clinical and epidemiologic data were collected at enrollment, and a stool sample was tested for enteropathogens by quantitative PCR. An algorithm was created based on the organism's cycle threshold (Ct) and association with MSD to identify the subset of *Cryptosporidium* PCR-positive (Ct <35) cases most likely to be attributed to MSD. Clinical outcomes were assessed at 2–3 months after enrollment.

**Results:**

One thousand one hundred six (22.9%) cases of MSD and 873 controls (18.1%) were PCR positive for *Cryptosporidium*; 465 cases (42.0%) were considered attributable to *Cryptosporidium*, mostly among children 6–23 months. *Cryptosporidium* infections peaked in The Gambia and Mali during the rainy season, while in Kenya they did not have clear seasonality. Compared with cases with watery MSD who had a negative PCR for *Cryptosporidium*, cases with watery MSD attributed to *Cryptosporidium* were less frequently dehydrated but appeared more severely ill using a modified Vesikari scale (38.1% vs 27.0%; *P* < 0.001), likely due to higher rates of hospitalization and intravenous fluid administration, higher prevalence of being wasted or very thin very thin (23.4% vs 14.7%; *P* < 0.001), and having severe acute malnutrition (midupper arm circumference <115 mm, 7.7% vs 2.5%; *P* < 0.001). On follow-up, *Cryptosporidium*-attributed cases had more prolonged and persistent episodes (43.2% vs 32.7%; *P* <0 .001) and linear growth faltering (change in height-for-age *z* score between enrollment and follow-up: −0.29 vs −0.17; *P* < 0.001).

**Conclusions:**

The burden of *Cryptosporidium* remains high among young children in sub-Saharan Africa. Its propensity to cause illness and further impact children longer term by compromising nutritional status early in life calls for special attention to enable appropriate management of clinical and nutritional consequences.

## Teaser

The burden of *Cryptosporidium* remains high among young children in sub-Saharan Africa. Its propensity to cause illness and further impact children longer term by compromising nutritional status early in life necessitates appropriate management of clinical and nutritional consequences.

The Global Enteric Multicenter Study (GEMS) examined a comprehensive panel of putative enteropathogens and demonstrated the relative importance of *Cryptosporidium* as a leading cause of moderate-to-severe diarrhea (MSD) and MSD-associated mortality across 7 sites in sub-Saharan Africa and South Asia [1]. Conducted prior to rotavirus vaccine introduction, GEMS (2007–2011) informed global estimates indicating that, each year, *Cryptosporidium* causes over 50 million diarrheal episodes and 48 000 deaths among children younger than 5 years, 88% of whom live in sub-Saharan Africa [2, 3]. Findings from GEMS supplemented previous observations that *Cryptosporidium* infections are linked to growth faltering and malnutrition [2, 4–7], which have been shown to increase the longer term risk of reduced physical fitness and diminished cognitive function [8]. Furthermore, malnutrition has been associated with an increased risk of *Cryptosporidium*-associated diarrhea [9], resulting in a vicious cycle of reinfection and repeated disease [10].

Since recommendations for universal use in infants in 2009 [11], rotavirus vaccines have been introduced in over 70% of Gavi-eligible countries, resulting in decreases in overall and rotavirus-specific diarrheal disease incidence and mortality [12, 13]. Given their impact on child health, rotavirus vaccines might be expected to alter the epidemiology of other enteropathogens. The Vaccine Impact on Diarrhea in Africa (VIDA) Study, conducted at 3 GEMS sites following introduction of rotavirus vaccine using methods comparable to GEMS, provides an opportunity to re-examine the prevalence, distinguishing clinical features, and impact of *Cryptosporidium* on child health (Kotloff et al., Unpublished data). Although no effective intervention for the prevention or treatment of cryptosporidial diarrhea has become available for public health use, therapeutics are under development. Therefore, to inform future case management, we also examined characteristics that might identify children with cryptosporidiosis who might be candidates for treatment in low-resource settings.

## METHODS

### Study Design and Objectives

VIDA was a population-based, age-stratified, matched case-control study of MSD among children 0–59 months of age living in Basse and Bansang, The Gambia; Bamako, Mali; and Siaya County, Kenya. The objectives of this analysis were to characterize episodes of MSD-attributed to *Cryptosporidium* to (1) describe the epidemiology by age, site, and season and (2) identify clinical characteristics that distinguish episodes of watery *Cryptosporidium*-attributable MSD from *Cryptosporidium* polymerase chain reaction (PCR)–negative episodes by examining the age, signs, symptoms, and seasonality at presentation for medical care. The overall design (Kotloff et al., Unpublished data) and statistical methods used in VIDA are detailed elsewhere [14], with key elements summarized below.

### Eligibility and Enrollment

VIDA cases and controls resided within each site's censused demographic surveillance system (DSS). During a 36-month period at each site, cases were eligible when they sought care at a sentinel health center (SHC) for a new and acute episode of diarrhea that met at least 1 of the following criteria for MSD: sunken eyes (more than usual per the caregiver), loss of skin turgor, dysentery, prescribed intravenous hydration, or a recommendation for hospital admission.

We aimed to enroll 8–9 eligible children per age stratum (infants, 0–11 months; toddlers, 12–23 months; and children, 24–59 months) each fortnight. For each MSD case, 1–3 controls without MSD were randomly selected from the DSS database; matched to the index case on sex, residence, and age [15]; and enrolled within 2 weeks of the index case.

### Clinical and Epidemiological Data Collection for Cases and Controls

The primary caregiver of each case and control provided demographic, epidemiological, and clinical information during a standardized enrollment interview. Each child underwent a focused physical examination that included measurement of height/length and mid-upper arm circumference (MUAC) [15]. Height/length measurements were converted to a *z* score based on World Health Organization (WHO) standards [16]. Caregivers recorded the occurrence of diarrhea daily for 14 days after enrollment using a pictorial memory aid [15]. At a single follow-up visit performed approximately 60 days postenrollment (acceptable range: 49–91 days), vital status was ascertained, anthropometric measurements were repeated, and the memory aid was reviewed and collected.

### Meteorological Data Collection

Both rainfall and temperature data routinely collected in The Gambia and Kenya were available for the study period. In The Gambia, government-operated meteorological stations in Basse and Bansang generate monthly summaries of both rainfall and the minimum, maximum, and average ambient temperature. In Kenya, stations located at Kisumu International Airport and operated by the meteorological department collected daily total rainfall and maximum and minimum ambient temperature. We were unable to obtain these data from a primary source for Bamako, Mali, and instead utilized an online source that models monthly averages using data collected from 1982 to 2012 [17].

### Laboratory Procedures

For this analysis, we tested a fresh stool sample passed by each case and the first matched control at enrollment by quantitative PCR (qPCR) using a custom TaqMan Array Card (TAC) that detected 26 enteropathogens, including the 18S rRNA gene of *Cryptosporidium* spp. [18]. Primers for the LIB13 locus, which differentiates *Cryptosporidium hominis* (the major pathogen for humans) from *Cryptosporidium parvum* (a subset of which causes diarrhea in humans), were included.

### Statistical Methods

Analysis of qPCR results to determine etiology have been described [14]. For the current analysis, stool samples were considered positive for an enteropathogen when the qPCR cycle threshold (Ct) value was less than 35. To assess whether a child's MSD was attributed to a specific pathogen, we first performed conditional logistic regression to measure the strength of the association between pathogen Ct and MSD status (case or control), adjusting for other enteric pathogens and allowing for effect modification by site and age stratum. For each MSD case, we then calculated the pathogen and episode-specific odds ratio (ORe) using an individual's pathogen Ct value. This ORe was converted to the episode-specific attributable fraction (AFe) using AFe = 1 – (1/ORe). An AFe ≥0.5, which translates to a pathogen-specific Ct value associated with a 2-fold higher likelihood of being an MSD case (vs a control), was considered “attributed.” This strategy was intended to minimize the inclusion of participants with subclinical infections. To identify a distinct clinical syndrome for cryptosporidiosis, we compared presenting features of children with *Cryptosporidium-*attributed watery (nonbloody) MSD with those with watery MSD that was not attributed to *Cryptosporidium*. We excluded cases who had a positive PCR for *Cryptosporidium* but who did not meet the criteria for attributable because we could not determine with certainty whether some of these children had attributable *Cryptosporidium*. Categorical variables were compared by the chi-square test or Fisher's exact test, as appropriate, and continuous variables were compared using a Wilcoxon rank-sum test. *P* values less than 0.05 was considered statistically significant.

To compare the severity of the clinical presentation of *Cryptosporidium*-attributed MSD with other etiologies of watery diarrhea, we used a 20-point modified Vesikari score (mVS) [19], which included all components of the original Vesikari score with some minor modifications (Supplementary Table 1). Stunting was defined as height/length-for-age *z* score (HAZ) less than –2 [20] and severe acute malnutrition was defined as MUAC less than 115 mm [21]. Weight was not used in the analysis because the impact of diarrheal dehydration could not be adequately quantified.

Seasonal patterns of *Cryptosporidium* were analyzed by estimating the total number of *Cryptosporidium*-attributed MSD cases seen at each site within each age stratum and for each month of the study. Accordingly, we calculated a site, age stratum, and calendar month–specific weight of the total number of children with diarrhea presenting at an SHC who met the criteria for MSD, divided by the total number of MSD cases enrolled. This weight was applied to the number of *Cryptosporidium*-positive MSD cases identified within that site, age stratum, and calendar month. The total estimated number of *Cryptosporidium*-attributed MSD cases for a given month is therefore taken as the sum over all age strata and all instances of that month within the study period.

The estimated total monthly *Cryptosporidium-*attributed MSD cases were plotted against the study period's average monthly temperature (monthly minimum plus the monthly maximum divided by 2) and the average monthly rainfall to qualitatively assess if *Cryptosporidium*-associated MSD has defined seasonal peaks at each site and if these peaks coincide with changes in temperature and rainfall. The rainy season for each site was defined as any month in which rainfall exceeded the site average over the study period.

### Ethical Review

This project was approved by the institutional review boards (IRBs) of the University of Maryland, Baltimore, Maryland, USA (HP-00062472); the US Centers for Disease Control and Prevention (CDC), Atlanta, Georgia, USA (reliance agreement, CDC protocol #6729); The Gambia Government/Medical Research Council/Gambia at the London School of Hygiene & Tropical Medicine (1409); the Comité d'Ethique de la Faculté de Médecine, de Pharmacie, et d'Odonto-Stomatologie, Bamako, Mali (no number); and the Kenya Medical Research Institute Scientific & Ethics Review Unit in Siaya County, Kenya (SSE 2996). Written, informed consent was obtained from the parent or primary caretaker of each child who met eligibility criteria before any research activities were performed.

## RESULTS

### *Cryptosporidium* Infections by Age and Site

Between 11 May 2015 and 23 July 2018, 4840 cases and 6213 matched controls were enrolled across the 3 study sites. A stool sample was tested for *Cryptosporidium* by qPCR from 4836 cases and 4836 first-matched controls, of which 1106 cases (22.9%) and 873 controls (18.1%) were positive for *Cryptosporidium*. Site differences in PCR positivity were seen, with Kenya generally having the lowest proportion among both cases and controls (Table 1).

**Table 1.** Proportion of Children With Moderate-to-Severe Diarrhea Who Tested Positive for *Cryptosporidium* Using qPCR, and Proportion Attributed to This Pathogen, by Subgroup Status

	The Gambia		Mali		Kenya	
	Cases	Controls	Cases	Controls	Cases	Controls
**0–11 months**						
No. tested by qPCR	540	539	595	595	585	585
No. (%) positive for *Cryptosporidium*	170 (31.5%)	127 (23.6%)	155 (26.1%)	124 (20.8%)	80 (13.7%)	67 (11.5%)
No. *Cryptosporidium*-attributed	91		83		43	
ȃȃȃȃPercentage attributed among those tested	16.9%		13.9%		7.4%	
ȃȃȃȃPercentage attributed among those PCR positive	53.5%		53.6%		53.8%	
**12–23 months**						
No. tested by qPCR	617	618	552	552	526	526
No. (%) positive for *Cryptosporidium*	166 (26.9%)	157 (25.4%)	141 (25.5%)	108 (19.6%)	115 (21.9%)	70 (13.3%)
No. *Cryptosporidium*-attributed	72		38		52	
ȃȃȃȃPercentage attributed among those tested	11.7%		6.9%		9.9%	
ȃȃȃȃPercentage attributed among those PCR positive	43.4%		27.0%		45.2%	
**24–59 months**						
No. tested by qPCR	520	520	461	461	440	440
No. (%) positive for *Cryptosporidium*	145 (27.9%)	116 (22.3%)	85 (18.4%)	72 (15.6%)	49 (11.1%)	32 (7.3%)
No. *Cryptosporidium*-attributed	47		19		20	
ȃȃȃȃPercentage attributed among those tested	9.0%		4.1%		4.5%	
ȃȃȃȃPercentage attributed among those PCR positive	32.4%		22.4%		40.8%	

“Positive” denotes qPCR cycle threshold <35. Abbreviations: MSD, moderate-to-severe diarrhea; PCR, polymerase chain reaction; qPCR, quantitative polymerase chain reaction.

**Figure 1.**
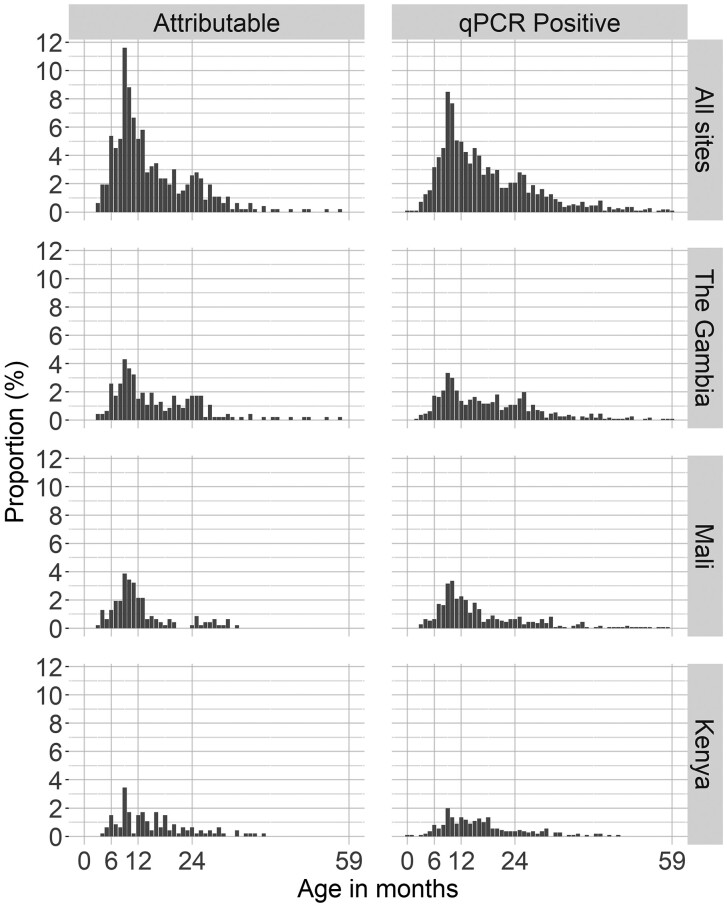
Distribution of attributable (AFe >0.5) and qPCR positive (Ct <35) *Cryptosporidium* MSD cases by age in months, separately for each study site and all sites combined in VIDA, 2015–2018. Abbreviations: AFe, episode-specific attributable fraction; MSD, moderate-to-severe diarrhea; qPCR, quantitative polymerase chain reaction; VIDA, Vaccine Impact on Diarrhea in Africa.

**Figure 2.**
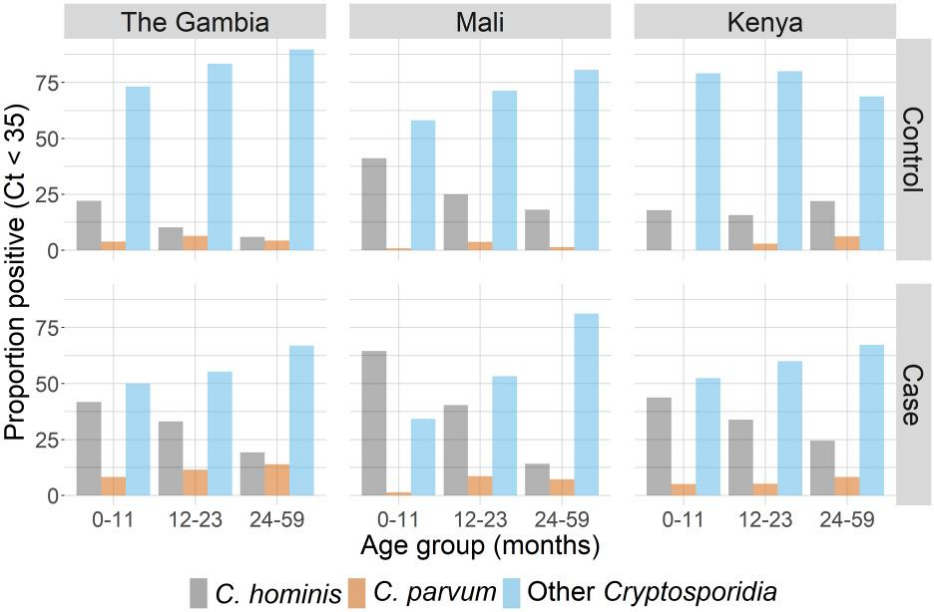
Proportion of *Cryptosporidium* MSD cases and controls who were PCR positive for *Cryptosporidium hominis*, *Cryptosporidium parvum*, or other *Cryptosporidia* by age group. Abbreviations: Ct, cycle threshold; MSD, moderate-to-severe diarrhea; PCR, polymerase chain reaction; VIDA, Vaccine Impact on Diarrhea in Africa.

Most (71.7%) MSD cases with *Cryptosporidium* detected by PCR were between 6 and 23 months of age, with a similar distribution of those considered to be attributable or not attributable (Figure 1), with the highest frequency between 6 and 11 months of age. PCR positivity among cases was more than 2-fold higher in the 6–11-month age group (363/1302 [27.9%]) than in infants younger than 6 months of age (42/419 [10.0%]), whereas detection was negligible after 3 years of age. Nearly 54% of PCR-positive *Cryptosporidium* episodes were considered attributable to *Cryptosporidium* among infants, with the proportion decreasing with age (Table 1). Age-related declines were also seen for the proportion of episodes associated with *C. hominis* but not for *C. parvum* or unspeciated *Cryptosporidium* (Figure 2).

### Clinical Findings

The most common presentation of the 465 children with *Cryptosporidium*-attributed MSD was watery diarrhea (94.4%) with vomiting (57.4%) and WHO-defined dehydration (92.8%); 61.1% were febrile (Table 2). Compared with the 3680 other episodes of watery MSD that had a negative PCR for *Cryptosporidium,* cases attributed to *Cryptosporidium* had significantly more vomiting (57.4% vs 46.8%; *P* < 0.001) and fever (61.1% vs 56.2%; *P* = 0.048) but significantly less severe dehydration by WHO criteria (*P* = 0.013). On the other hand, cases with *Cryptosporidium* were more likely to receive intravenous fluids (*P* <0 .001) and to be admitted to the hospital (*P* = 0.064), and have, overall, a significantly higher mVS (*P* < 0.001). A distinguishing feature was a higher prevalence of appearing wasted or very thin (23.4% vs 14.7%; *P* <0 .001) and of severe acute malnutrition (7.7% vs 2.7%; *P* <0 .001) among *Cryptosporidium*-attributed cases than in other children with watery MSD (*P* < 0.001) (Table 2).

**Table 2.** Clinical Presentation of Attributable *Cryptosporidium* Moderate-to-Severe Diarrhea Cases Compared With All Other Watery Diarrhea

	*Cryptosporidium*-Attributed MSD (n = 465)	All Watery MSD^a^ (n = 3680)	*P*
**Demographic features**			
Age, median (IQR), months	12 (9, 20)	15 (9, 25)	<0.001*
ȃSex, n (%)			
Female	204 (43.9%)	1724 (46.8%)	0.245
ȃȃMale	261 (56.1%)	1956 (53.2%)	
**Findings at enrollment**			
ȃNo. of median (IQR)diarrhea days prior to SHC visit	3 (2–4)	3 (2–3)	<0.001*
ȃMental status, n (%)			
ȃȃNormal	355 (76.3%)	2608 (70.9%)	0.048*
ȃȃRestless/irritable	96 (20.6%)	936 (25.4%)	
ȃȃLethargic/unconscious	14 (3%)	136 (3.7%)	
ȃFever (temperature >38°C or parental perception), n (%)	284 (61.1%)	2068 (56.2%)	0.048*
ȃVomiting, n (%)	267 (57.4%)	1722 (46.8%)	<0.001*
ȃSunken eyes, more than normal, n (%)	449 (96.6%)	3580 (97.3%)	0.458
ȃOral mucosa, n (%)			
ȃȃNormal	92 (19.8%)	734 (19.9%)	0.996
ȃȃSomewhat dry	351 (75.5%)	2774 (75.4%)	
ȃȃVery dry	22 (4.7%)	172 (4.7%)	
ȃSkin pinch, n (%)			
ȃȃNormal	319 (68.6%)	2542 (69.1%)	.551
ȃȃSlow return	141 (30.3%)	1114 (30.3%)	
ȃȃVery slow return	5 (1.1%)	24 (0.7%)	
ȃWHO-defined dehydration, n (%)			
ȃȃNone	33 (7.1%)	175 (4.8%)	0.013*
ȃȃSome	380 (81.7%)	2955 (80.3%)	
ȃȃSevere	52 (11.2%)	550 (14.9%)	ȃ
ȃModified Vesikari score, n (%)	ȃ	ȃ	ȃ
ȃȃMild	109 (23.4%)	1205 (32.8%)	<.001*
ȃȃModerate	179 (38.5%)	1479 (40.2%)	ȃ
ȃȃSevere	177 (38.1%)	992 (27.0%)	ȃ
ȃȃMedian (IQR)	9 (7, 11)	8 (6,11)	<.001*
ȃStunted, n (%)	ȃ	ȃ	ȃ
ȃȃHAZ ≥ –2	357 (76.8%)	2867 (77.9%)	0.621
ȃȃHAZ < –2	108 (23.2%)	813 (22.1%)	ȃ
ȃUndernourished (wasted/very thin), n (%)	109 (23.4%)	541 (14.7%)	<0.001*
ȃSevere acute malnutrition, n (%)	ȃ	ȃ	
ȃMUAC ≥115 mm	429 (92.3%)	3581 (97.3%)	<.001*
ȃMUAC <115 mm	36 (7.7%)	99 (2.7%)	ȃ
ȃAbnormal hair (sparse, loose, straight), n (%)	26 (5.6%)	111 (3%)	.005*
ȃ“Flaky paint” appearance of the skin, n (%)	5 (1.1%)	20 (0.5%)	.191
ȃRequired rehydration, n (%)	ȃ	ȃ	ȃ
ȃȃNo	8 (1.7%)	3 (0.1%)	<0.001*
ȃȃYes, oral alone	403 (86.7%)	3396 (92.3%)	ȃ
ȃȃYes, oral and IV	54 (11.6%)	281 (7.6%)	ȃ
ȃAdmitted to the hospital, n (%)	33 (7.1%)	182 (5.0%)	0.064
**Findings at follow-up**	n = 445 (95.7%)	n = 3538 (96.1%)	ȃ
ȃTotal no. of days with diarrhea	6 (4–9)	5 (3–8)	<.001*
ȃDiarrhea duration stratum, n (%)	ȃ	ȃ	ȃ
ȃȃAcute (1–6 days)	253 (56.9%)	2380 (67.3%)	<.001*
ȃȃProlonged (7–13 days)	137 (30.8%)	835 (23.6%)	ȃ
ȃȃPersistent (≥14 days)	55 (12.4%)	323 (9.1%)	ȃ
ȃChange in HAZ	−0.29 (−0.52, 0.00)	−0.17 (−0.42, 0.03)	<0.001*
ȃUndernourished (wasted/very thin), n (%)	6 (1.3%)	234 (6.6%)	0.337
ȃMUAC <115 mm, n (%)	4 (0.9%)	26 (0.7%)	0.320
ȃStunted, n (%)	ȃ	ȃ	ȃ
ȃȃHAZ ≥ –2	332 (71.4%)	2749 (74.7%)	0.128
ȃȃHAZ < –2	133 (28.6%)	931 (25.3%)	ȃ
ȃDied, n (%)	6 (1.3%)	26 (0.7%)	0.162

Statistically significant. Abbreviations: HAZ, height-for-age *z* score; IQR, interquartile range; IV, intravenous, MSD, moderate-to-severe diarrhea; MUAC, mid-upper arm circumference; PCR, polymerase chain reaction; SHC, sentinel health center; WHO, World Health Organization.

Episodes with positive PCR for *Cryptosporidium* that did not meet criteria for attributed were excluded from this analysis.

When clinical outcomes at the 60-day follow-up visit were compared, *Cryptosporidium*-attributed cases were more likely than *Cryptosporidium*-negative cases to experience prolonged or persistent diarrhea (43.2 vs 32.7%; *P* < 0.001). Despite having a comparable frequency of stunting at enrollment, they exhibited significantly more linear growth faltering (Δ HAZ: −0.29 vs −0.17; *P* <0 .001) at follow-up (Table 2). They also had a higher mortality, but the difference was not statistically significant (1.3% vs 0.7%; *P* = 0.162).

### Seasonality

**Figure 3.**
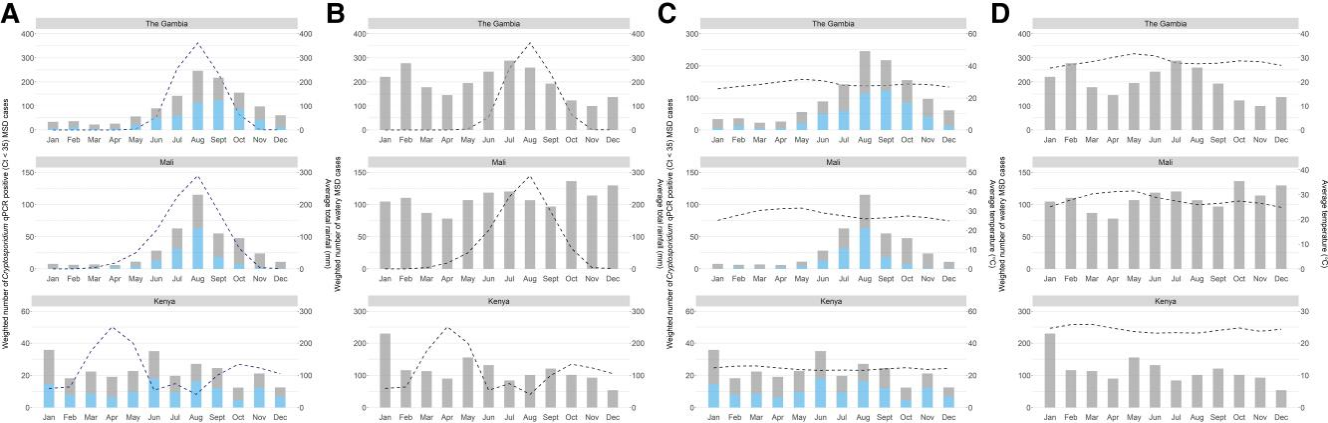
*A*, Number of monthly qPCR positive (Ct <35) *Cryptosporidium* MSD cases (gray) and number of attributable (AFe >0.5) *Cryptosporidium* MSD cases (blue), both weighted by site and age group, and the monthly average rainfall (mm, dashed line) by study site in VIDA, 2015–2018. *B*, Number of monthly watery MSD cases, weighted by site and age group, and the monthly average rainfall (mm, dashed line) by study site in VIDA, 2015–2018. *C*, Number of monthly qPCR positive (Ct <35) *Cryptosporidium* MSD cases (gray) and number of attributable (AFe >0.5) *Cryptosporidium* MSD cases (blue), both weighted by site and age group, and the monthly average temperature (°C, dashed line) by study site in VIDA, 2015–2018. *D*, Number of monthly watery MSD cases, weighted by site and age group, and the monthly average temperature (°C, dashed line) by study site in VIDA, 2015–2018. Abbreviations: AFe, episode-specific attributable fraction; Ct, cycle threshold; MSD, moderate-to-severe diarrhea; qPCR, quantitative polymerase chain reaction; VIDA, Vaccine Impact on Diarrhea in Africa.

*Cryptosporidium* cases followed a seasonal pattern, with a peak in the rainy season from June to October in The Gambia and Mali when temperatures tended to be lower (Figure 3). During the seasonal rainy season peaks in Mali and The Gambia, the proportion of watery MSD among children 6–23 months of age presenting to the SHC with attributable *Cryptosporidium* was 49.2% in The Gambia and 86.5% in Mali (Table 3). Even without an obvious seasonal pattern at the Kenya study site, where temperatures remain steady and a major and minor rainy season typically occurs, the proportion of watery MSD among children 6–23 months of age with attributable *Cryptosporidium* was 34% in the rainy season.

**Table 3.** Moderate-to-Severe Diarrhea (MSD) Cases 6–23 Months of Age Attributed to *Cryptosporidium* (AFe ≥0.5) But Who Did Not Have Dysentery and Watery MSD Cases 6–23 Months of Age, by Season and Site

	The Gambia			Mali			Kenya		
	Cryptosporidium Attributed	All Watery	*P*	Cryptosporidium Attributed	All Watery	*P*	Cryptosporidium Attributed	All Watery	*P*
No. with watery MSD	128	641		111	877		85	731	
Rainy season^a^	63 (49.2%)	154 (24.0%)	<.001	96 (86.5%)	285 (32.5%)	<.001	29 (34.1%)	296 (40.5%)	.6194

Data are presented as n (%) unless otherwise indicated. Abbreviation: AFe, episode-specific attributable fraction.

Rainy season was defined as months with higher-than-average rainfall during the study period. In The Gambia these months were July to September, in Mali they were June to September, and in Kenya there were 2 periods—namely, March to May and September to November.

## DISCUSSION

An association of *Cryptosporidium* diarrheal disease with subsequent malnutrition and increased mortality among infants and children living in low- and middle-income countries (LMICs) has long been recognized [2, 4–6], but differences in study design, populations, and diagnostic assays among studies did not allow comparisons of the magnitude of this effect. The use of standardized methods over 3 years in GEMS, across multiple sites in 2 continents, provided evidence supporting the widespread nature of a *Cryptosporidium* MSD disease burden that included an increased risk of associated stunting and mortality [1, 7, 22, 23]. In VIDA, using comparable methods at GEMS sites in Mali, The Gambia, and Kenya, we found that nearly a decade later, the disease burden and adverse outcomes associated with *Cryptosporidium* persist [18, 22].

Numerous impediments have hindered efforts to develop effective tools to control *Cryptosporidium*. The ability to curb transmission through improvements in sanitation and hygiene is stymied by the ease of transmission (a low infectious inoculum, prolonged shedding, and persistence of viable oocysts in the environment) and resistance to heat and chlorination [24]. The lack of robust animal models, molecular genetic tools, and culture systems has impeded the development of drugs and vaccines [4, 7]. Indications that vaccination might be an effective preventive strategy include suggestions of age-related acquisition of immunity in LMICs [25–27] and observations that mucosal antibodies confer protection in infants [28, 29]. However, to date, no vaccine has been evaluated in a clinical trial. Only 1 drug, nitazoxanide, has shown efficacy in children from LMICs, but numerous barriers prevent implementation [30]. Fortunately, a new pipeline of promising drug candidates is undergoing preclinical evaluation [31–33]. It is likely that implementation of a therapeutic in LMICs will require a simple and inexpensive point-of-care diagnostic to identify children who might benefit from targeted therapy [34]. In the absence of an assay, we attempted to determine whether *Cryptosporidium*-attributed MSD episodes could be distinguished from other episodes of watery MSD on clinical grounds. Although a discrete syndrome was not apparent, we found that the combination of being aged 6–23 months and presenting for medical attention for MSD in the rainy season would select 49–87% of attributable cases, depending on the site. Thus, every child with cryptosporidiosis treated would require treating 2.4, 3.0, and 10.2 uninfected children in The Gambia, Mali, and Kenya, respectively.

Despite appearing less dehydrated, the most common complication of watery diarrhea, we found that infants and young children with *Cryptosporidium*-attributed MSD had poorer outcomes than other children with watery MSD during 2–3 months postenrollment. They were more likely to experience prolonged and persistent diarrheal episodes, had significantly more linear growth faltering, and were almost twice as likely to die (1.3% vs 0.7%) during the 2–3-month follow-up period. Although the mortality difference was not statistically different with the small sample sizes available, it is consistent with the effect size in other reports [1, 35]. The poor outcome may be related to the propensity for *Cryptosporidium* infection to persist and to affect vulnerable children at a young age, particularly those with wasting who might be intolerant of recommended case management [2, 33]. In contrast, children with rotavirus who reach a health center and receive life-saving rehydration therapy have reduced mortality [23, 36]. Our findings emphasize the need to identify and provide extended support to children with *Cryptosporidium*-attributed MSD, and to better understand the pathogenic mechanisms and the response to clinical and nutritional management to guide more effective interventions. Given the complexity of implementing point-of-care therapeutic strategies, the feasibility of primary prevention using drugs and vaccines also warrants consideration [37, 38].

There was a distinct seasonal peak of *Cryptosporidium*-associated MSD in the rainy season in The Gambia and Mali. A similar trend has been observed in GEMS and in many countries across sub-Saharan Africa [39–42], South Asia [40], and Latin America [43, 44] in both urban [45] and rural settings [40, 46]. A possible mechanism is that surface water contaminated by feces accumulates and is used to drink, cook, prepare foods, bathe, and swim [47]. In Kenya, where seasonality was less clear, *Cryptosporidium* exposure might be minimized during the rainy season due to the use of rain as the main source of drinking water, which may be less likely to be contaminated [48].

A limitation of our study is that we were unable to measure underlying comorbidities such as human immunodeficiency virus (HIV), which could result in nutritional faltering while also representing a known risk factor for *Cryptosporidium*. The low HIV prevalence in The Gambia and Mali and the observation that the lowest proportion of *Cryptosporidium*-attributed MSD was found in Kenya (which had the highest burden of HIV) suggest that HIV was not a major underlying risk factor for *Cryptosporidium* in our population. Furthermore, a GEMS substudy in Kenya found no association between *Cryptosporidium* and either HIV infection or HIV exposure [48]. The case-control design prohibited us from measuring risk factors—for example, the degree to which acute malnutrition at enrollment was the result of *Cryptosporidium* MSD rather than a cause of it. The use of a highly sensitive PCR and then selecting a subset most likely to represent *Cryptosporidium*-attributed MSD potentially could have resulted in the misclassification of cases who had low pathogen burdens that were actually causing their MSD. We mitigated this effect by excluding children with PCR-positive, unattributable MSD from our comparisons with watery diarrhea.

### Conclusions

*Cryptosporidium* continues to be a major cause of MSD in sub-Saharan Africa, predominantly affecting the 6–23-month-old age group. Its predilection to infect and further disable nutritionally compromised children early in life calls for special attention to ensure the identification and aggressive management of the clinical and nutritional consequences. Supporting the development of preventive and therapeutic interventions should be prioritized to avoid the lifelong risks for poor cognitive and physical health outcomes and premature mortality [4, 7]. The high prevalence of *Cryptosporidium* in the rainy season suggests that seasonal treatment might be a feasible strategy should an effective treatment be identified.

## Supplementary Data

Supplementary materials are available at *Clinical Infectious Diseases* online. Consisting of data provided by the authors to benefit the reader, the posted materials are not copyedited and are the sole responsibility of the authors, so questions or comments should be addressed to the corresponding author.

## Supplementary Material

ciad044_Supplementary_Data

## Notes

***Acknowledgments***. The authors express their deep gratitude to the families who participated in these studies, the clinical and field staff for their exceptional hard work and dedication, and to the physicians, administration, and health officials at every site who generously provided facilities and support for the conduct of the study. They are grateful to Catherine Johnson, Chris Focht, and Nora Watson at the Emmes Company, LLC, for expert data management and reporting. Special thanks go to Carl Kirkwood, Duncan Steele, and Anita Zaidi at the Bill & Melinda Gates Foundation for helpful oversight, Kathy Neuzil for thoughtful suggestions, and to the following members of our International Scientific Advisory Committee for providing insightful comments and guidance: Janet Wittes (Chair), George Armah, John Clemens, Christopher Duggan, Stephane Helleringer, Ali Mokdad, James Nataro, and Halvor Sommerfelt.

***Financial support*.** This study was funded by the Bill & Melinda Gates Foundation grants OPP1111236 and OPP1116751.

***Supplement sponsorship*.** This supplement was sponsored by the Bill & Melinda Gates Foundation. This study is based on research funded in part by grants from the Bill & Melinda Gates Foundation (OPP1111236/OPP1116751).
