# Supplementary material for: Clinical and Epidemiologic Features of Cryptosporidium-Associated Diarrheal Disease Among Young Children Living in Sub-Saharan Africa: The Vaccine Impact on Diarrhea in Africa (VIDA) Study
Source: Clin Infect Dis. 2023 Apr 19;76(Suppl 1):S97–S105. doi: 10.1093/cid/ciad044 (PMC10116562; doi:10.1093/cid/ciad044)
Supplement: ciad044_Supplementary_Data [file ciad044_supplementary_data.docx]

# Title: Clinical and epidemiologic features of *Cryptosporidium-*associated diarrheal disease among young children living in sub-Saharan Africa: The Vaccine Impact on Diarrhea in Africa (VIDA) study

**Authors:** M. Jahangir Hossain, Helen Powell, Samba O. Sow, Richard Omore, Anna Roose_,_ Joquina Chiquita M. Jones, Syed M.A. Zaman, Henry Badji, Golam Sarwar, Irene N. Kasumba, Uma Onwuchekwa, Sanogo Doh, Alex O. Awuor, John B. Ochieng, Jennifer R. Verani, Jie Liu, Sharon M. Tennant, Dilruba Nasrin, Leslie P. Jamka, Stephen R. C. Howie, Martin Antonio, Eric R. Houpt, and Karen L. Kotloff

| **Table S1. Comparison of Vesikari Score and the modified Vesikari Score (mVS) used in VIDA** | | | | | | |
| --- | --- | --- | --- | --- | --- | --- |
| **Parameter** | **Score** | | | | | **Comment** |
|  | **Vesikari** | | | | |  |
|  | **1** | **2** | | | **3** |  |
| Diarrhea |  |  | | |  |  |
| Max. no. stools/day | 1-3 | 4-5 | | | >6 |  |
| Duration (days) | 1-4 | 5 | | | >6 |  |
| Vomiting |  |  | | |  |  |
| Max. no. emesis/day | 1 | 2-4 | | | >5 |  |
| Duration (days) | 1 | 2 | | | >3 |  |
| Temperature (°C) | 37.1-38.4 | 38.5-38.9 | | | >39.0 |  |
| Dehydration | N/A | 1-5% or some | | | >6% or severe | . |
| Treatment | Rehydration | Hospitalization | | | N/A | Participants who are “hospitalized” for at least 24 hours OR who receive IV therapy are considered “hospitalized” and receive a corresponding score of 2 points for this parameter |
|  | **VIDA Modified Vesikari Score (mVS)** | | | | |  |
| Diarrhea |  | |  |  | |  |
| Max. no. stools/day | 3 | | 4-5 | >6 | | 1-2 days not permissible; definition of diarrhea requires >3 stools/day |
| Duration (days) | 1-4 | | 5 | 6-7 | | Cannot exceed 7; enrolment criteria requires <7 days |
| Vomiting |  | |  |  | |  |
| Max. no. emesis/day | 1 | | 2-4 | >5 | |  |
| Duration (days) | 1 | | 2 | >3 | | May be truncated because of diarrhea duration enrolment criterion |
| Temperature (°C) | 37.1-38.4 | | 38.5-38.9 | >39.0 | | No change |
| Dehydration | N/A | | some | severe | | No change |
| Treatment | Rehydration | | Hospitalization/IV | N/A | | No change |
